# Hybrid Materials of Bio-Based Aerogels for Sustainable Packaging Solutions

**DOI:** 10.3390/gels10010027

**Published:** 2023-12-28

**Authors:** Urška Vrabič-Brodnjak

**Affiliations:** Department of Textiles, Graphic Arts and Design, Faculty of Natural Sciences and Engineering, University of Ljubljana, Snežniška 5, 1000 Ljubljana, Slovenia; urska.vrabic@ntf.uni-lj.si; Tel.: +386-1-200-32-89

**Keywords:** bio-based aerogels, hybrid packaging materials, sustainability, eco-friendly packaging

## Abstract

This review explores the field of hybrid materials in the context of bio-based aerogels for the development of sustainable packaging solutions. Increasing global concern over environmental degradation and the growing demand for environmentally friendly alternatives to conventional packaging materials have led to a growing interest in the synthesis and application of bio-based aerogels. These aerogels, which are derived from renewable resources such as biopolymers and biomass, have unique properties such as a lightweight structure, excellent thermal insulation, and biodegradability. The manuscript addresses the innovative integration of bio-based aerogels with various other materials such as nanoparticles, polymers, and additives to improve their mechanical, barrier, and functional properties for packaging applications. It critically analyzes recent advances in hybridization strategies and highlights their impact on the overall performance and sustainability of packaging materials. In addition, the article identifies the key challenges and future prospects associated with the development and commercialization of hybrid bio-based aerogel packaging materials. The synthesis of this knowledge is intended to contribute to ongoing efforts to create environmentally friendly alternatives that address the current problems associated with conventional packaging while promoting a deeper understanding of the potential of hybrid materials for sustainable packaging solutions.

## 1. Introduction

Sustainable packaging presents a critical aspect of modern industry, addressing environmental concerns and consumer demands. It plays a central role in today’s industrial landscape, and it is the answer to increasing environmental concerns and changing consumer expectations. Conventional packaging materials, which are predominantly derived from non-renewable sources, contribute significantly to environmental degradation. Therefore, bio-based packaging, a fast-growing market, is driven by the need for sustainability and technological innovation [1]. The global bio-based packaging market was valued in 2022 at USD 7.92 billion, and it is expected to grow to around USD 25.86 billion by 2032, growing at a compound annual growth rate of 12.56% during the forecast period from 2023 to 2032 [2]. 

To improve bio-based packaging, nanostructures of biopolymers and hybrid solutions are being researched [3]. These hybrid packaging materials are made from renewable raw materials and have improved performance in terms of strength, heat resistance, and barrier properties [4,5,6,7,8]. They can also offer functionalities such as antimicrobial, antifouling, and sensing properties, making them suitable for active and intelligent packaging [9,10,11,12,13]. The need to address the sustainability, biodegradability, and environmental safety of conventional inorganic aerogels has fueled scientific efforts to explore environmentally friendly alternatives [14]. Characterized by their porous, lightweight structure, these aerogels represent an environmentally friendly alternative that is in line with the principles of sustainable development. The use of bio-based aerogel packaging materials is in line with the need for long-term global sustainability and the transition to a low-carbon economy. They utilize renewable resources and environmentally friendly manufacturing processes to deliver biodegradable and safe products of high quality and functionality [1,2,3,4,5,6,7,8,9,10,11,12,13,14,15].

Bio-based aerogels are derived from various renewable sources such as sugar cane, vegetable oils, proteins, starches, chitosan, alginate, pectin, lignin, cellulose, and proteins, which have been shown to be useful in the production of aerogels [16,17,18,19,20,21,22,23,24,25]. These aerogels have special properties that make them well suited for packaging applications and in bioengineering [26,27,28,29,30]. Recent studies in this field have led to advances that shed light on their diverse applications, improved properties, and innovative synthesis methods. Researchers have investigated alternative feedstocks for bio-based aerogels to broaden their sources and enhance sustainability [9,10,11,14]. Studies have explored the use of waste materials, agricultural by-products, and unconventional sources to synthesize aerogels, aligning with the principles of a circular and green economy [16,17,18]. Recent research has also focused on adapting the properties of bio-based aerogels to specific application requirements. This has included modifying the mechanical strength, thermal conductivity, and porosity of the aerogel through innovative processing techniques, resulting in materials with improved performance characteristics [20,21,22,23,24,25,26,27,28,29,30,31,32,33,34,35,36,37,38,39,40,41].

The potential of bio-based aerogels for sustainable packaging has not yet been sufficiently researched in the packaging industry. Despite the increasing recognition of bio-based aerogels as sustainable packaging materials, there is a recognizable research gap in comprehensively exploring their potential in this area. The full range of opportunities and challenges associated with the integration of bio-based aerogels into packaging materials should be addressed. Bridging this research gap is therefore essential to unlock the true potential of these materials and advance sustainable packaging technologies.

The main objectives of this review manuscript contain the following:

(a) Investigation of the fabrication methods of hybrid materials composed of various bio-based aerogels for sustainable packaging:
  This research aims to explore the different manufacturing methods for the synthesis of hybrid materials composed of different bio-based aerogels. The summary includes techniques in which bio-based aerogels are fused with other materials to form hybrid composites in order to optimize their properties for sustainable packaging applications.
(b) Eco-friendliness and sustainability benefits of hybrid materials in packaging:
  Another focus of this study is the comprehensive assessment of the environmental friendliness and sustainability benefits associated with hybrid materials containing bio-based aerogels.
(c) Potential applications and highlighting their contributions to sustainable packaging practices:
  Beyond the laboratory bench, the research aims to explore the practical applications of hybrid materials for sustainable packaging. By identifying and illustrating potential uses, from improving insulation properties to increasing barrier functionality, these materials will be positioned as transformative agents in the pursuit of sustainable packaging practices.

## 2. Types of Hybrid Bio-Based Aerogel Materials for Sustainable Packaging

**Figure 1:**
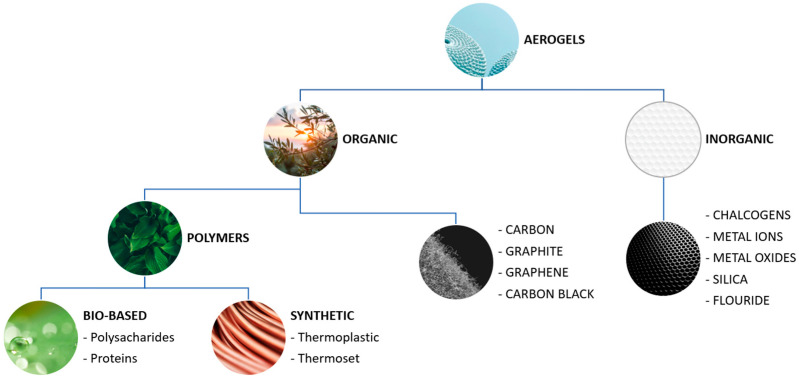
Classification of aerogels according to used raw materials [16,17,18,19,20].

Aerogels can be divided into two categories, namely organic and inorganic aerogels [16]. Each of these categories is divided into subcategories according to the type of materials and the structure of the gel (Figure 1) [16,17,18,19,20].

Bio-based polymers are gaining ground in many areas, and the use of these compounds is a suitable alternative to reduce the environmental impact of certain packaging (e.g., food packaging) [17,18,19]. The attractiveness of these materials is usually related to the synthesis routes of biopolymer aerogels. Their potential to be customized through precise strategies gives them certain properties that suit their specific uses [20,21,22,23,24,25].

Bio-based aerogels have special properties that are well suited for packaging and also for biomedical purposes, such as applications in tissue engineering, regenerative medicine, and drug delivery [26,27,28,29,30]. As mentioned, such aerogels derived from renewable sources represent a promising class of materials with multiple applications.

This review analyses different types of the most commonly used bio-based aerogels (polysaccharide (cellulose, chitosan, alginate, starch)-, protein-, lignin- and pectin-based aerogels) and highlights their sources and selection criteria based on environmental friendliness and properties.

### 2.1. Polysaccharide-Based Aerogels

Polysaccharides, complex carbohydrates consisting of repeating sugar units, are at the forefront of interdisciplinary research due to their diverse and multi-faceted applications. As fundamental components of biological systems, polysaccharides play crucial roles in the cell structure, energy storage, and material processes and production. Polysaccharides are known for their ability to self-assemble into certain physical structures, which has already been explored by many researchers in the formation of aerogels, xerogels, and cryogels [31,32,33,34,35,36]. The group of potential polysaccharide aerogels includes cellulose, chitosan, alginate, pectin, and starch. Their properties such as porosity, low weight, and a large surface area improve their suitability for various applications, especially for drug delivery [37,38,39].

In connection with the use of polysaccharides for the production of aerogels, many of the further described bio-based polysaccharide aerogels in the field of packaging have been investigated and researched for their properties such as biodegradability, biocompatibility, non-toxicity, bioactivity, and environmental friendliness [40,41,42,43,44,45].

#### 2.1.1. Cellulose-Based Aerogels

Cellulose, a ubiquitous biopolymer found in plant cell walls, serves as an important starting material for bio-based aerogels. The synthesis of cellulose-based aerogels involves a careful process aimed at utilizing the unique properties of cellulose. The extraction of cellulose involves a variety of sources, including various plants and plant-derived materials such as rice straw, hemp, cotton, wood, potatoes, and bagasse [46,47,48,49]. The complex performance characteristics of cellulose, including degree of polymerization, size, crystallinity, and thermal stability, are closely related to the plant species and extraction processes, which include pretreatment, post-treatment, and comminution [5,13,49,50]. This relationship influences the structure and performance of cellulose aerogels. While cellulose can be synthesized from bacterial cultures (bacterial nanocellulose) such as *Acetobacter xylinum*, resulting in higher crystallinity (80%) and the absence of impurities such as lignin and hemicellulose, the in vitro synthesis of low molecular weight cellulose is possible through cellulase catalysis or ring-opening polymerization [46]. In addition, cellulose derivatives such as carboxymethyl cellulose, cellulose esters, and cellulose ethers are available via grafting, sulphonation, and TEMPO-mediated oxidation, utilizing the high chemical reactivity of the hydroxyl groups in each glucose unit of the cellulose chain [51,52]. There are many reviews that comprehensively address the structure, properties, and applications of cellulose and its derivatives [53,54,55,56,57]. The mechanical properties and moisture affinity of aerogel materials are significantly improved by the use of cellulose and its derivatives [57,58,59]. The advantages of using cellulose as an aerogel precursor are manifold: there is an infinite and renewable supply of cellulose raw material; the abundance of hydroxyl groups in the cellulose chain eliminates the need for crosslinking agents, thus simplifying the aerogel manufacturing process; and the chemical modification of cellulose facilitates the improvement of the mechanical strength and structural properties of cellulose aerogels. The categorization of cellulose aerogels based on raw materials leads to three different groups [60]: natural cellulose aerogels (nanocellulose aerogels, bacterial cellulose aerogels), regenerated cellulose aerogels, and cellulose-derived aerogels.

It is well known that cellulose-based aerogels have exceptional properties that make them well suited for sustainable packaging applications. With a surface area of 200 to 1000 m^2^/g and a porosity of often more than 90%, these aerogels have an extensive network of interconnected cellulose fibrils [57,58,59,60,61]. This intricate structure contributes to their remarkable insulating properties and makes them extremely effective in thermoregulation. The mechanical strength of cellulose-based aerogels is remarkable, which is due to the robust network of cellulose fibers. This property is particularly advantageous in packaging applications where durability and resistance are of paramount importance. The light weight of these aerogels makes them even more attractive as it offers them a good balance between strength and lower material density. Hybrid PVA/cellulose/nanocellulose aerogels show promising properties for the controlled release of bioactive compounds in food systems, which could benefit bioactive packaging structures, as explored by de Oliviera [15]. Cellulose-based aerogels have attracted considerable attention due to their renewable and biocompatible properties. These aerogels can be produced from various sources such as fruit waste [46], cellulose nanocrystals (CNCs) [47], and NaOH/urea solution [48].

They have an ultra-low density, high porosity, and low thermal conductivity, which makes them suitable for heat-insulating applications [49,50]. Aerogels containing cellulose nanocrystals from rice and oat hulls have been proven to be water absorbers for food packaging and show promising industrial applications in various fields [44,45,62].

The hierarchical structure of cellulose-based aerogels enables the incorporation of nanoparticles, which increases their multifunctionality. There is a third generation of aerogels: nanocellulose-based aerogels, which are based on abundant and sustainable cellulose as a raw material [63,64,65,66,67]. These aerogels seamlessly combine the traditional aerogel properties such as high porosity and large specific surface area with the exceptional properties of cellulose. Currently, nanocellulose aerogels have proven to be a fascinating platform for various functional applications in different fields, including adsorption, separation, energy storage, thermal insulation, electromagnetic interference shielding, and biomedical applications [51,52,53,54,55,56].

In addition, the cellulose concentration and the drying method can influence the micromorphology and crystalline structure of aerogels. The introduction of flame-retardant particles such as zinc borate improves the thermal stability and flame retardancy of cellulose aerogels [61,66,67]. These materials have the potential for various applications including oil spill treatment, energy storage, actuator development, and packaging [66,67]. The use and modification of cellulose-based aerogels offers a wide range of possibilities in the field of materials science and technology.

The environmental friendliness of cellulose-based aerogels is based on the renewable nature of the cellulose sources. The cellulose obtained from plant-based raw materials ensures the sustainable life cycle of the aerogels. In addition, the biodegradability of cellulose meets the environmental protection goals and addresses concerns associated with the disposal of packaging materials. The biodegradability of cellulose-based aerogels meets the increasing demand for environmentally friendly packaging solutions. With their complex synthesis process and outstanding properties, cellulose-based aerogels offer a glimpse into the future of sustainable packaging materials. The combination of environmental friendliness, insulating properties, and mechanical strength makes cellulose-based aerogels promising candidates for overcoming the environmental challenges associated with conventional packaging.

#### 2.1.2. Chitosan-Based Aerogels

Chitosan-based aerogels are produced through a careful synthesis process that begins with the deacetylation of chitin from crustacean shells. This process converts chitin into chitosan, a biopolymer with a wide range of applications. The chitosan is then dissolved in a suitable solvent, often acetic acid, resulting in a viscous solution. Gelation is brought about using methods such as freeze drying or supercritical drying, which promotes the development of a three-dimensional porous structure characteristic of aerogels [67,68]. The resulting aerogels have a network of interconnected pores, which contributes to their light weight and porous nature [67,68,69,70].

Chitosan-based aerogels have a number of properties that make them highly suitable for sustainable packaging applications [71,72,73]. In particular, their inherent biocompatibility makes them safe for direct contact with food, which is a crucial aspect in the packaging of consumer goods. Aerogels also have exceptional antibacterial properties, a property that can extend the shelf life of packaged goods by inhibiting microbial growth. In addition, chitosan-based aerogels have a large surface area and porosity, which improves their heat-insulating properties. This combination of properties makes them versatile materials that are suitable for different packaging requirements.

The mechanical strength of chitosan-based aerogels is remarkable and provides a robust framework for potential packaging applications [74,75,76,77]. The interconnected network of chitosan molecules contributes to the structural integrity of the material and ensures a long shelf life under various packaging conditions. This tensile strength increases the versatility of chitosan-based aerogels as they can withstand the rigors of transportation and handling while maintaining the integrity of the packaged contents [69,78].

The environmentally friendly profile of chitosan-based aerogels is emphasized by the fact that they are derived from a by-product of the fishing industry. The use of crustacean shells, which would otherwise be considered waste, is in line with the principles of sustainability and resource efficiency. In addition, the biodegradability of chitosan ensures that these aerogels have a minimal impact on the environment at the end of their life cycle and are a more environmentally friendly alternative to conventional packaging materials. Chitosan-based aerogels exhibit remarkable compatibility with various materials, which facilitates their integration into hybrid composites. The ability to combine chitosan-based aerogels with other substances opens up opportunities to tailor the properties of the resulting hybrid materials to the specific requirements of sustainable packaging. This versatility in hybridization expands the range of potential applications for chitosan-based aerogels in the packaging industry. Their unique combination of biocompatibility, antibacterial properties, mechanical strength, and environmental friendliness make them versatile materials that can make an important contribution to promoting environmentally conscious packaging practices.

#### 2.1.3. Alginate-Based Aerogels

The synthesis of alginate-based aerogels requires a careful process to utilize the unique properties of these marine-derived polysaccharides. Sodium alginate is extracted from marine algae, usually brown algae, via a series of alkaline treatments such as with Na_2_CO_3_ [79,80,81,82]. After extraction, the sodium alginate is mixed with a crosslinking agent, often calcium ions, to effect gelation [78,79,80]. The resulting gel is then subjected to supercritical drying, a critical step that transforms the gel into a porous aerogel structure while preserving its intricate network.

Alginate-based aerogels have several properties that make them attractive for sustainable packaging [81,82,83,84,85]. The inherent biocompatibility of alginate enables safe contact with food, making these aerogels suitable for food packaging. Their high water absorbency is advantageous for scenarios where moisture resistance is critical for maintaining the quality of the packaged goods [82,83]. The porous structure of alginate-based aerogels contributes to their exceptional insulating properties, which effectively protect temperature-sensitive products during storage and transport. The environmentally friendly profile of alginate-based aerogels stems from the renewable nature of the algae that serve as the primary source of alginate [83,84,85,86]. Seaweed is abundant, grows quickly, and does not compete with food crops for arable land, which is in line with sustainable sourcing practices. In addition, the biodegradability of alginate ensures that these aerogels have a minimal impact on the environment and provide a responsible solution for the disposal of packaging materials at the end of their life cycle. They demonstrate versatility in packaging applications. Their compatibility with a wide range of substances, including liquids and solids, enables a variety of packaging solutions [86,87,88]. Whether as a coating material to extend the shelf life of fruit or as an insulating layer for temperature-sensitive pharmaceuticals, alginate-based aerogels demonstrate their adaptability and effectiveness for a variety of packaging requirements. The synthesis of hybrid materials via incorporating alginate-based aerogels into composite structures opens up new opportunities for innovation. By combining alginate with other materials such as cellulose or polymers, the properties of the resulting hybrid aerogels can be customized to meet specific requirements as researched by Zhang et al. [89].

Composite aerogels made from bamboo shoots, cellulose, and sodium alginate have been shown to have the potential for sustainable, biocompatible drug delivery, with potential applications in dietary supplements and cosmetics. Hugo et al. presented crosslinked aerogels made from cellulose nanofibers and alginate that enable the rapid, continuous, and large-scale production of porous, lightweight materials for energy storage, mechanical strain, and humidity sensors.

Such materials have the potential to be used not only for energy storage but also for other applications [90]. Most research has been conducted in the biomedical field, where alginate-based aerogels have been used for bone regeneration, wound healing, tissue engineering, etc. [91,92,93]. The synthesis and properties of alginate-based aerogels emphasize their potential as versatile and environmentally friendly materials for sustainable packaging.

#### 2.1.4. Starch-Based Aerogels

Starch, one of the various natural polysaccharides, has gained increasing attention in research as a material for the production of aerogels, and promises diverse applications in various fields [94,95,96,97,98,99,100]. It is non-allergenic, non-toxic, and generally recognized as being safe, as well as being abundant and inexpensive. These properties make starch-based aerogels particularly attractive and well suited for nutritional and food applications [95,96,97]. Starch-based aerogels can be produced in a variety of shapes and dimensions, including monoliths, films, and microspheres ranging from nanoscale to micron sizes. Starch-based aerogels represent a remarkable class of bio-based materials that have significant potential for sustainable packaging applications. The synthesis of starch-based aerogels typically involves the extraction of starch from renewable sources such as corn, wheat, potato, or cassava [94,95,96]. The extracted starch is then mixed with a suitable solvent such as epichlorohydrin and glutaraldehyde. It reacts with the hydroxyl groups in the starch, causing crosslinking and forming a gel. Gelation is triggered by processes such as freeze drying or subcritical drying. This leads to the formation of a three-dimensional porous network, which is characteristic of aerogel structures. Starch-based aerogels have several key properties that make them attractive for sustainable packaging, such as biodegradability, versatility, and low cost, and they are a renewable resource [97,98,99,100].

These aerogels often exhibit high porosity and provide a large surface area that can be beneficial for various functions, including absorption and insulation. The crosslinked network of starch molecules in the aerogel structure contributes to its mechanical stability, making it suitable for applications where durability is important. The use of starch as a raw material for aerogels is in line with sustainability goals as starch sources are renewable and widely available. The biodegradability of starch-based aerogels further enhances their eco-friendly profile and minimizes their environmental impact throughout their life cycle. One potential application for starch-based aerogels is food packaging. By incorporating lignocellulosic nanofibrils, the water absorption of waxy maize-starch-based aerogels could be reduced from 15 g/g to 12 g/g, as shown by Ago et al. [101]. The mechanical properties of the resulting composite aerogel are comparable to those of polystyrene foam. This composite aerogel is therefore a promising environmentally friendly and sustainable alternative for packaging. Starch-based aerogels containing agar or microcrystalline cellulose also have the potential to be used for the controlled release of active ingredients, as an absorbent, and as a source of resistant starch [102]. On the other hand, aerogel based on konjac glucomannan/starch enriched with wheat straw has a high potential for thermal insulation due to its low thermal conductivity and good thermal stability [103]. In the context of sustainable packaging, where the focus is on reducing the environmental footprint, starch-based aerogels are a compelling solution.

#### 2.1.5. Pectin-Based Aerogels

Pectin, which consists of α-(1–4)-linked D-galacturonic acid residues and is composed of homogalacturonan and rhamnogalacturonan, has at least 17 different monosaccharides in its structure [104]. The behavior of pectin in solutions is determined by the ratio of methylated or amidated groups to non-modified galacturonic acid, which in turn influences the properties of the resulting pectin-based materials [104,105,106,107]. The model explains the mechanism of the crosslinking reaction with divalent metal ions, whereby the crosslinks are formed by divalent ions occupying electronegative cavities in the bifurcated band structure of the carboxyl groups [106]. Pectin-based aerogels derived from pectin, a complex polysaccharide found in plant cell walls, represent a unique class of bio-based aerogels. The synthesis process usually starts with the extraction of pectin from citrus fruits, apples, or other plant sources [104,105]. The extracted pectin is then dissolved in water. The water-soluble components of plant cell walls, including pectin, can be released and extracted using a water-based extraction process. Gelation is initiated through methods such as freeze drying or supercritical drying. This process leads to the formation of a three-dimensional network, resulting in the porous and light structural characteristics of aerogels. Amidated pectins, which are characterized by a low methyl ester content, can form gels over a wide pH range in the presence of divalent cations. In addition, the introduction of alcohols, such as ethanol or tert-butanol, enhances the hydrophobic interactions between the pectin chains, resulting in higher mechanical strength for the hydrogels [107,108]. In contrast, the presence of amide groups, as observed in low-methylated, non-amidated pectins, leads to the formation of gels with better mechanical properties [107,109]. As researched by Tkalec et al., the ethanol-induced gelation of pectin, alginate, xanthan gum, and guar gum accelerates the production of aerogels that have a large surface area and are suitable for life-science applications [110]. These aerogels have special properties that make them interesting for various applications, including sustainable packaging. With their inherent biocompatibility and biodegradability, these aerogels are in line with environmentally conscious principles. The porous structure contributes to their low weight and therefore offers advantages in packaging, where weight reduction is important. In addition, pectin-based aerogels can have unique mechanical properties that are influenced by the specific pectin sources, allowing for versatility to meet different packaging requirements. The molecular arrangement within the aerogel matrix contributes to variations in mechanical strength, flexibility, and porosity, making pectin-based aerogels adaptable to specific packaging requirements.

The ability of pectin to absorb water makes these aerogels suitable for packaging applications where moisture control is critical. Hong-Bing et al. found that aerogels made from pectin and clay derived from renewable sources showed accelerated biodegradation compared to wheat starch. The addition of clay and polyvalent catalysts further increased the biodegradation rates [111]. The biodegradability of pectin is in line with the growing demand for environmentally friendly packaging materials and helps reduce the environmental impact.

### 2.2. Protein-Based Aerogels

Proteins exhibit a high degree of complexity and have a sophisticated supramolecular chemistry that offers fascinating possibilities for material production. The current review takes specific properties, including the self-assembly of proteins into fibrils and the propensity of proteins or protein-derived materials to form gels, as notable examples of their valuable properties [112,113,114,115]. Protein-based aerogels derived from natural proteins such as soya, whey, or silk represent a compelling category in the field of bio-based aerogels. The synthesis process involves the extraction of proteins from sustainable sources, followed by dissolution in a suitable solvent. Gelation is usually induced by methods such as freeze drying or supercritical drying, which enables the formation of a three-dimensional aerogel structure [112,113,114,115]. The resulting protein-based aerogels exhibit a porous and crosslinked network that reflects their aerogel nature. The inherent biocompatibility of proteins makes these aerogels safe for contact with food and makes them viable candidates for food packaging. Depending on the protein source, these aerogels can have different mechanical properties, ranging from flexibility to robustness, offering great versatility for packaging solutions. Researchers have used protein nanofibrils that were effectively combined with gelatin to create aerogels with enhanced mechanical properties [113,115].

The application of mechanochemical processing has allowed the manipulation of gelling behavior and provided an environmentally friendly and scalable method to tune the properties and functionality of protein-based aerogels. This is a simple way to produce non-toxic and biodegradable aerogel materials with favorable mechanical strength [113]. The use of proteins as a raw material for aerogels is in line with sustainability goals due to their renewable nature. Proteins of plant or animal origin offer a biodegradable alternative to conventional packaging materials, addressing concerns about environmental impact and waste. The environmentally friendly profile of protein-based aerogels also extends to their potential for the circular economy, emphasizing the importance of responsible material use and disposal.

### 2.3. Lignin-Based Aerogels

Lignin, a complex and heterogeneous biopolymer derived from plant cell walls, has attracted attention as a sustainable starting material for aerogels. The synthesis of lignin-based aerogels involves the extraction of lignin from lignocellulosic biomass such as wood or agricultural residues [116,117,118]. Various methods, including dissolution in ionic liquids or other suitable solvents, are used to produce a homogeneous lignin solution, which is the prerequisite for subsequent gelation. The gelling process is often facilitated through freeze drying or supercritical drying. The structural properties of lignin-based aerogels are influenced by the lignin source. Different plant species and processing methods result in lignin with different molecular weights, compositions, and functionalities. This diversity gives these aerogels a range of mechanical properties and allows them to be customized for specific applications. The lignin-rich composition contributes to the unique structural subtleties observed in these aerogels. Such aerogels have special properties that make them promising materials for various applications including sustainable packaging. These aerogels often have a porous structure with a large surface area, which contributes to their low weight. The interconnected lignin networks within the aerogel skeleton provide mechanical stability, making them suitable for applications where both strength and flexibility are important. As Cantu et al. presented in their research, lignin-based aerogels can be produced from wheat straw via crosslinking with oligo (alkylene glycol) diglycidyl ethers and offer the potential for greater value-added utilization in chemical synthesis [116].

Due to their versatile properties, lignin-based aerogels have been produced from bacterial cellulose/lignin-based carbon aerogels in a catalyst-free, low-cost process, and these are suitable for flexible solid-state energy storage and other applications [117]. In addition, organosolv lignans from various lignocellulosic biomasses (aspen, pine, and barley straw) could be used to produce highly porous lignin-5-methylresorcinol-formaldehyde aerogels with a large surface area and high pore volume [118]. The inherent UV-blocking properties of lignin make these aerogels potential candidates for the protection of packaged goods against light-induced deterioration [118].

The use of lignin as a raw material for aerogels is in line with sustainability goals, as it is abundant in nature and is a by-product of various industries. The biodegradability of lignin-based aerogels ensures a minimized environmental footprint throughout their life cycle. In addition, the reuse of lignin from industrial processes such as pulp and paper production contributes to the circular economy by transforming a waste product into a valuable and sustainable material.

As described in the subchapters, various organic, bio-based aerogels, including cellulose, chitosan, starch, lignin, protein, and pectin, have different structural characteristics (Table 1). Overall, cellulose-based aerogels often have a fibrous network, while chitosan-based aerogels have an amorphous structure with inherent antibacterial properties. Such aerogels are known for their thermal insulation, while starch-based aerogels have structural variations influenced by the arrangement of the starch molecules and offer versatile functionality due to their porosity. Lignin-based aerogels, which are derived from plant cell walls, have a unique composition. Protein-based aerogels vary in structure depending on the protein source, and pectin-based aerogels show structural variations depending on the pectin source and extraction method. This diversity enables customization to specific packaging requirements, with the mechanical properties of the bio-based aerogels varying. Cellulose-based aerogels exhibit excellent mechanical strength due to their fibrous nature, while chitosan-based aerogels offer flexibility and robustness, making them particularly suitable for specific packaging requirements, and they also have antibacterial properties. Starch-based aerogels, which are influenced by the arrangement of the starch molecules, have mechanical properties due to their porosity. Lignin-based aerogels have unique mechanical properties due to their lignin content. Protein-based aerogels have versatile mechanical properties depending on the protein source, and pectin-based aerogels offer flexibility depending on the pectin source and extraction method. Understanding these mechanical variations is critical for selecting aerogels tailored to specific packaging applications.

Biodegradability is a common feature of bio-based aerogels that come from renewable sources, such as the mentioned bio-based aerogels that meet sustainability goals. Their inherent biodegradability ensures responsible disposal at the end of their life, contributing to the circular economy. This eco-friendly profile makes them attractive alternatives to traditional packaging materials from non-renewable sources. Understanding these functional properties enables the strategic selection of aerogels for specific sustainable packaging applications. The versatility of bio-based aerogels opens up possibilities for various sustainable packaging applications. Cellulose-based aerogels offer robustness and thermal insulation. Chitosan-based aerogels with antibacterial properties are suitable for packaging perishable goods. Starch-based aerogels offer flexibility for different packaging requirements. Lignin-based aerogels meet specific packaging requirements due to their unique composition, and pectin-based aerogels are suitable for moisture-sensitive products. This versatility allows these materials to be strategically integrated into a range of sustainable packaging solutions.

The comparative analysis emphasizes the diversity of structural, mechanical, biological, functional, and application-related aspects of bio-based aerogels. Understanding these differences is crucial for informed decision making in the selection and design of sustainable packaging materials tailored to specific industry needs and environmental concerns.

## 3. Fabrication Hybrid Bio-Based Aerogel Materials for Sustainable Packaging

Research into hybrid materials, achieved by combining different bio-based aerogels, represents a pioneering achievement in sustainable packaging research. This innovative approach utilizes the unique properties of the individual aerogels to achieve synergistic effects, improve overall performance, and solve specific packaging problems.

Typically, the processes for obtaining bio-based aerogels involve the fusion of precursors, subsequent gelation, and, most importantly, the removal of pore-filling solvents from the wet gels without significantly reducing the volume or densifying the network. This is usually achieved by converting the pore-filling solvent into a supercritical fluid and gradually releasing it as a gas. With this method, aerogels can retain the structural configuration of their wet gel precursors [14]. Therefore, the formation of aerogels consists of three steps, the first being the dispersion of the biopolymer-based precursor solution (sol), the second being the gelation process (gel), and the third being the drying of the wet gel.

It has been confirmed that the gelling process is the most critical step; therefore, the production methods for gelling methods include [14]:

- Sol-gel coacervation;
- Heating and cooling;
- Crosslinking using chemicals or enzymes;
- High shear, pH, and salt;
- Emulsion gelation;
- Internal gelation (ultrasound);
- Ethanol-induced gelation;
- Others.

**Figure 2:**
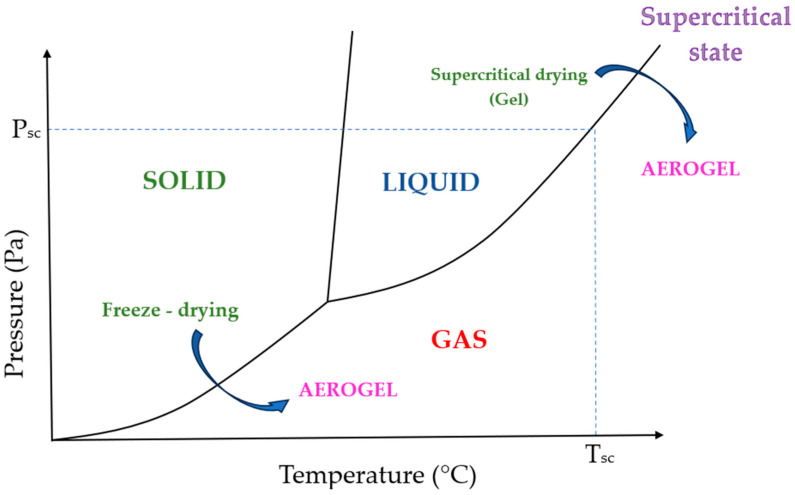
Phase diagram of different drying methods of pure compounds for aerogel preparation [119].

Figure 2 shows two methods for the transition from gel to aerogel, characterized by the solid–gas transition, which shows the transition from a frozen gel to a dried porous gel during freeze drying. The transition from a liquid to a gas during supercritical drying requires an increase in temperature and pressure (curved arrow) so that the phase boundary between liquid and gas is not exceeded. This transition to the supercritical range eliminates the surface tension and capillary forces.

Researchers have proposed three common strategies for the production of drying methods, namely supercritical drying, freeze drying, and ambient pressure drying, as well as microwave drying [118,119,120,121,122,123,124,125,126,127,128,129,130].

### 3.1. Supercritical Drying Method

The supercritical drying process prevents the collapse of the gel structure and maintains the high porosity characteristic of aerogels. The unique properties of supercritical fluids, such as their adjustable density and low surface tension, make them ideal for this application. In this process, the wet gel is heated in a closed vessel until the temperature and pressure exceed the critical values of the liquid trapped in the pores of the gel [119,131]. This leads to a state in which the liquid and vapor phases are no longer distinguishable from each other, eliminating the capillary forces. After the gas has been released and the material has cooled down, the aerogel is removed from the autoclave. Under supercritical conditions, the surface tension between liquid and gas is no longer present as there are no longer any liquid–gas interfaces. The use of supercritical carbon dioxide for drying protects the gel structure and results in materials with minimized shrinkage, a reduced pore size, and a larger specific surface area [132]. This method preserves the nanometer-sized features and pores and occasionally achieves thermal conductivities below those of air. However, a notable disadvantage of supercritical drying is that it is very time-consuming [119,132]. In addition, significant amounts of solvents and the use of relatively expensive supercritical gases contribute to increased manufacturing costs and potential environmental impacts [119]. The specific conditions for supercritical drying vary depending on the solvent; water, for example, requires a critical temperature of approximately 374 °C and a critical pressure of approximately 22 Pa [119]. The supercritical drying of various gels such as agar, alginate, chitosan, and cellulose continues to be developed, combining renewable raw materials with environmentally friendly carbon dioxide processing. Gawryla et al. presented an alternative approach using sublimation instead of supercritical drying. An aqueous cellulose dispersion is produced, followed by a solvent exchange with tert-butanol and finally supercritical drying [133]. As noted by Subrahmanyam et al., carbon dioxide pressurized to 5 MPa has also been used for the ionic crosslinking of amidated pectin [122]. The resulting aerogels exhibited high porosity with low density, high specific surface area, and considerable pore volume [121]. In the context of using biopolymers for aerogel formation and maximizing their potential, numerous studies have been conducted to reduce production times and costs and to facilitate the scalable production of aerogels [123].

This process has several advantages as it ensures the uniform removal of the liquid phase without capillary forces, prevents the aerogel structure from collapsing, and maintains its high porosity. The use of supercritical fluids enables faster drying times compared to conventional methods and thus contributes to a higher efficiency in the production process. It is a scalable process suitable for large-scale production, which makes it interesting for industrial applications and the commercialization of bio-based aerogels. However, the gentle nature of supercritical drying minimizes structural damage to the aerogel, resulting in improved mechanical strength and durability. At the same time, various approaches to simplify the manufacturing processes have been proposed in order to reduce the cost of aerogels.

### 3.2. Freeze Drying Method

Supercritical drying has been replaced by more economical and environmentally friendly methods such as freeze drying or drying at ambient pressure [124,125,126,127]. Bio-based aerogels produced through freeze drying have unique structural and morphological properties. The interconnected pore network, with sizes ranging from nanometers to micrometers, contributes to the exceptional surface area and porosity of the aerogel. This method involves several important steps, including gelation, freezing, and sublimation. The precursor solution is first converted into a gel, whereby the three-dimensional network structure is retained. The subsequent freezing of the gel leads to the formation of ice crystals within the structure. In the final step, the frozen water is removed via sublimation, leaving behind a highly porous aerogel structure.

Bio-based aerogels produced through freeze drying have unique structural and morphological properties. The interconnected pore network, with sizes ranging from nanometers to micrometers, contributes to the exceptional surface area and porosity of the aerogel. The resulting structures, known as cryogels, have different properties compared to aerogels. Cryogels generally have a higher density and lower surface area, with a porosity of up to 80% and only half the internal surface area of aerogels [128,129]. This discrepancy is primarily due to the formation of large ice crystals during the development of the gel network in the freezing process. This leads to an increased number of macropores and volume shrinkage. In contrast to aerogels synthesized via hot or vacuum drying, cryogels exhibit less shrinkage and a narrower distribution of pore sizes.

The freeze drying process offers several advantages and represents an uncomplicated, cost-effective, and environmentally friendly technique. The use of water as a solvent combined with the simplicity of the drying process contributes to its attractiveness. In addition, this method can be applied to bio-based polymers such as starch, lignin, pectin, alginate, gelatin, and cellulose [119,130,131,132,134]. However, the disadvantages include a prolonged processing time; volume changes during freezing, which can lead to the collapse of the aerogels; and the relatively high energy consumption. The resulting networks have a micrometric thickness and microscopic spacing and exhibit better thermal and mechanical properties compared to conventional polymer foams.

### 3.3. Ambient Pressure Drying Method

Drying methods under ambient pressure are an attractive alternative to supercritical drying as no extreme pressure conditions are required. In this approach, the temperature and relative humidity are carefully controlled during the drying process so that the solvent (e.g., acetone, ethanol) can be gradually removed without compromising the structural integrity of the aerogel. The ambient pressure drying method is promising for bio-based aerogels as it minimizes energy consumption and facilitates production scalability. The ambient pressure drying method offers several advantages for the production of bio-based aerogels, including lower energy consumption, simplified equipment requirements, and a potentially more cost-effective production process. However, challenges such as longer drying times and the need for the precise control of ambient conditions need to be addressed in order to optimize the method for different bio-based feedstocks [130].

### 3.4. Microwave Drying Method

In the synthesis of aerogels, microwave drying methods are used to produce materials characterized by a large surface area and desirable porosity. The aerogels produced using this method have similar structures to those produced through freeze drying, but with a prevalence of smaller, interconnected macropores. In particular, this method proves to be more time-saving and provides promising results in a much shorter time frame. It is based on the selective heating of polar molecules in the material, whereby the heat is generated directly in the substance. This targeted and rapid heating shortens drying times compared to conventional methods. The uniform distribution of microwave energy throughout the material ensures the efficient removal of water or solvents, resulting in the formation of aerogels with the desired properties. In addition, this method is a faster technique with promising results, as presented by Liang Wang et.al [135]. They demonstrated that microwave-crosslinked bio-based starch/clay aerogels exhibited higher biodegradability and improved mechanical properties compared to poly(vinyl alcohol)-based aerogels. A comparison of the individual drying methods for the production of aerogels and their properties is shown in Table 2.

When improving durability, it is important to maintain a balance with environmental aspects. The choice of crosslinking agents and reinforcing materials should be guided by environmentally friendly principles to ensure that the hybrid bio-based aerogels remain sustainable throughout their life cycle. Green crosslinking agents and biodegradable reinforcing materials contribute to the overall environmental sustainability of these hybrid materials.

## 4. Properties and Applications of Packaging for Hybrid Bio-Based Aerogel Materials

The combination of different bio-based aerogels requires strategic hybridization strategies in order to exploit the strengths of the individual components. These may include blending cellulose and chitosan to obtain a composite aerogel that has both fibrous starch and antibacterial properties. Alternatively, a mixture of starch- and protein-based aerogels could provide a balanced solution that takes into account the water-absorbing capabilities of the starch and the mechanical properties of the proteins. The choice of aerogel components depends on the desired properties for specific packaging applications.

Crosslinking is a crucial step in the hybridization process that promotes the cohesion of the various bio-based aerogels within the hybrid matrix [119,131,132,133,134,135,136]. Common crosslinking agents include glutaraldehyde, epoxides, and diisocyanates. In cellulose–chitosan hybrids, for example, glutaraldehyde can facilitate crosslinking by forming covalent bonds between the hydroxyl groups of the cellulose and the amino groups of the chitosan. The crosslinking process improves the mechanical strength and stability of the hybrid material and ensures that the synergies between the aerogels are utilized effectively.

Reinforcement techniques are critical for strengthening the structural integrity of hybrid bio-based aerogels, especially in the context of sustainable packaging, where durability is of paramount importance. Nanoparticle reinforcement is one such technique wherein nanoparticles such as silicon dioxide or graphene are incorporated into the aerogel matrix. This reinforcement improves mechanical strength, thermal stability, and barrier properties. In hybrid aerogels, for example, the combination of cellulose with silica nanoparticles could result in a material with improved strength and heat resistance that is ideal for packaging applications [133,135].

The fusion of bio-based aerogels through crosslinking and reinforcement techniques results in hybrid materials that outperform the individual components in terms of durability and performance. The crosslinked bonds form a robust network that prevents the hybrid structure from disintegrating under mechanical stress. Reinforcement, particularly with nanoparticles, provides additional strength and barrier properties that ensure the hybrid material can withstand environmental conditions and preserve the quality of the packaged goods.

The characterization of hybrid bio-based aerogel materials requires a multi-faceted approach to understand their physical, chemical, and structural properties (Table 3). It includes the analysis of the structure, morphology, thermal stability, and mechanical properties of hybrid bio-based aerogels.

The packaging properties of hybrid bio-based aerogel materials are evaluated in terms of barrier properties, mechanical strength, flexibility, and compatibility with different product types. These properties are critical in determining the effectiveness of a material in various packaging applications and its ability to replace conventional packaging materials without compromising performance. The environmental friendliness of hybrid bio-based aerogels is assessed, taking into account factors such as raw material sourcing, energy consumption during synthesis, and the overall carbon footprint. Biodegradability studies will analyze the degradation of such materials under different environmental conditions, simulating scenarios that occur in landfills or in nature.

An environmental impact assessment analyzes the life cycle of hybrid bio-based aerogels, taking into account raw material sourcing, synthesis processes, and disposal at the end of the life cycle. Comparative analyses with conventional packaging materials provide information on the general environmental friendliness. Biodegradability studies under simulated environmental conditions aim to understand the degradation behavior and validate the potential of aerogels to alleviate the problems associated with long-life packaging waste. The text emphasizes how the environmental friendliness and biodegradability of these aerogels contribute significantly to reducing the environmental footprint of packaging materials.

Thermal conductivity analyses show the heat transfer properties of the hybrid bio-based aerogels, which are characterized by low thermal conductivity and improved insulating properties.

The exceptional thermal insulation properties of these aerogels play a crucial role in advanced temperature control during transport and storage. Mechanical tests, including tensile, compression and bending tests, evaluate the strength, elasticity, and durability of aerogel-based packaging. Comparative studies with conventional packaging materials emphasize the benefits of aerogel-based solutions in terms of mechanical performance, which contribute to improved product protection and reduced consumption of packaging material (Table 3) [86,87,88,89,90,91,92,93,94,95,96,97,98,99,100,101,102,103,104,105,106,107,108,109,110,111,112,113,114,115,116,117,118,119,120,121,122,123,124,125,126,127,128,129,130,131,132,133,134,135,136,137,138,139,140,141,142,143,144,145,146,147,148,149,150,151,152,153].

**Table 3:** Properties of hybrid bio-based aerogels for different packaging applications.

Hybrid Bio-Based Polymers/Aerogels	Fabrication Method	Properties	Properties of the Material	Applications	References
Aldehyde and carboxyl nanocellulose and crosslinked carboxymethyl chitosan	Freeze drying technique using liquid nitrogen	Porosity: 98.8%	Maintains adsorption capacity in dye solutions over a wide pH range, allowing them to be regenerated and be successively reused for at least six cycles	Ultralight green functional materials	[138]
*Arundo donax* biomass and extract from *Arundo donax*	Sol-gel, freeze drying	Density: 10.21–14.39 mg∙cm^−3^ Water vapor sorption: 0.39–0.91 g/g	Reduced oxidation processes	Active food packaging	[145]
Alginate/ lignin, starch, pectin, carrageenan, methyl and carboxymethyl cellulose, gellan gum, and gelatin	Sol-gel; hydrogel, wherein gel is frozen using liquid nitrogen	Density: 0.017 g∙cm^−3^ Pore volume: 2.3–9.5 cm^3^∙g^−1^ for pore sizes < 150 nm	Excellent thermal insulation	Active packaging; tissue and bone engineering	[83]
Alginate, pectin	Sol-gel, freeze drying	Porosity: 65.60–70.00% Bulk density: 0.1923–0.6158 g∙m^−1^∙L^−1^	High porosity (>65.60%) with thermal stability over 140 °C; high flexibility	Active packaging	[146]
Bleached cellulose fibers and cellulose nanoparticles	Freeze drying technique using liquid nitrogen	Specific surface area: 143–162 m^2^∙g^−1^ Diameter of pores: 5–13 nm	Improved thermal conductivity and mechanical properties.	Different products for thermal insulation; also used for food packaging	[131]
Cellulose whisker, PVA, Clay	Freeze drying technique using liquid nitrogen	Density: 0.01–0.101 g∙cm^−3^ Compression modulus: 18–788	Increased tensile strength, enhanced mechanical properties	Packaging products for filled polymer-like properties	[120]
Cellulose/lignin	Supercritical CO_2_ drying	Density: 0.025–0.114 g∙cm^−3^ Specific surface area: 108–539 m^2^∙g^−1^	Completely opaque and shining white; a brownish color is increased with the amount of lignin in the polymer mix	Packaging products with nanofibrillar aerogel by changing the polymer mix	[133]
Cellulose-NaOH solution and softwood kraft fibers	Supercritical CO_2_ drying	Specific surface area: 340 m^2^∙g^−1^ Compressive modulus: 8.2 MPa	Very good adhesion between the kraft fibers and the matrix; non-porous fibers decrease the specific surface area	Versatile porous materials used as separators	[119]
Citrus pectin, cellulose nanofibers	Freeze drying technique using liquid nitrogen	Density: 0.109–0.122 g∙cm^−3^ Porosity: 90.37–98.11%	Increased compression and tensile stress in the aerogel; good water vapor adsorption/release performance	Active packaging—edible-fungus-moisture-regulating packaging	[147]
CMC/CNF	Freeze drying technique using liquid nitrogen	Density: 0.05–0.109 g∙cm^−3^ Porosity: 93.19–96.84% Compression modulus: 1000–8700 kPa Thermal conductivity: <54 mW m^−1^ K^−1^	Increased modulus and strength; low thermal conductivities; low densities	Thermal insulating packaging materials; reinforcing agent for biocomposite materials	[136]
Corn starch, agar, microcrystalline cellulose	Sol-gel, hydrogel-alcogel, supercritical CO_2_ drying	Porosity: 90–95% Young’s modulus: 0.38–5.39 Water absorption: 200–300% Aqueous stability: 7 days	Mechanical reinforcement; higher specific surface in comparison to pure starch aerogels	Active packaging	[148]
Glucose, albumin	Sol-gel, hydrogel-frozen, freeze drying	Surface area: 247–476 m^2^∙g^−1^ Pore volume: 0.38–0.7 cm^3^ g^−1^ Porosity: 95–97% Pore diameter: <100 nm	Several binding sites available for the covalent modification and attachment of bioactive substances	Active packaging	[149]
Maize starch, calcium alginate, flavanoid Quercetin	Sol-gel, hydrogel-alcogel; supercritical CO_2_ drying	Surface area: 70–80 m^2^∙g	Increased migration barrier when Quercetin is added; supercritical adsorption is suitable to obtain controlled-release systems to be used as an packaging active layer	Active food packaging	[150]
Maleic acid grafted CNF	Freeze drying technique using liquid nitrogen	Density: 0.0112–0.0315 g∙cm^−3^ Specific surface: 19.5 m^2^∙g^−1^ Compression modulus: 1000–8700 kPa	Good network stability in water and springiness after compression	Reinforcing agent for biocomposite and packaging materials	[137]
Microcrystalline cellulose and lignin	Supercritical CO_2_ drying	Density: 0.1–0.135 g∙cm^−3^ Specific surface area: 200 m^2^∙g^−1^	Cellulose and lignin are not compatible in the NaOH	Used separately as aerogel materials not as a mixture	[140]
Microfibrillated cellulose, kapok	Sol-gel, hydrogel-frozen, freeze drying	Density: 5.1 mg∙cm^−3^ Porosity: 99.58% Oil sorption capacity: 130.1 g/g Hydrophobicity: 140.1°	Increased mechanical strength, specific properties can be easily adapted	Used as oleogels for edible and active packaging	[152]
Potato starch, konjac, glucomannan, wheat straw powder, gelatin	Sol-gel, freeze drying	Density: 0.043 g∙cm^−3^ Porosity: 94.5% Thermal conductivity: 0.046–0.053 W/mK	High specific surface area composed of large continuous mesopores, meso- and macroporous transport structure	High end applications (e.g., sensing, charge storage and fast adsorption processes); filtration and packaging materials	[141]
PVA/cellulose/nanocellulose aerogels	Sol-gel; freeze drying	Crystallinity index: 48.8–61.4%	Increased water capacity, sustained release of the agar-based extract in food simulants	Active, food packaging materials	[15]
Soy protein, nanocellulose	Sol-gel, hydrogel-alcogel, supercritical CO_2_ drying	Density: 0.19–0.25 g∙cm^−3^ Surface area: 384–478 m^2^∙g, Porosity: 84–88% Thermal conductivity: 0.033 w∙m^−1^∙K^−1^	Low-cost, with term storage stability; antioxidant properties	Active packaging and food packaging needed for thermal insulation	[151]
Starch, cellulose, essential oil: *Thymus daenensis* Celak	Sol-gel, freeze drying	Density: 18.42–54.77 g∙cm^−3^ Porosity: 64–87%	Increasing the starch content results in a denser cellulose aerogel and a significant reduction in aerogel porosity; a gradual inhibitory effect of the aerogels on psychrophiles and yeast–mold populations in cheese has been noted	Antimicrobial packaging for dairy products	[153]
TEMPO-CNF	Freeze drying technique using liquid nitrogen	Density: 0.0017–0.0081 g∙cm^−3^ Porosity: 95.5–99.9% Specific surface area: 10.9 m^2^∙g^−1^	Ultra-lightweight, highly porous, superior wet compressibility, and complete shape recovery	Amphiphilic super-absorbents for selective oil removal and recovery	[139]
Whey protein, egg white protein, sodium caseinate aerogels	Sol-gel, hydrogel-alcogel, supercritical CO_2_ drying	Loading capacity: 63% (*w*/*w*)	Antibacterial properties, high processing versatility toward chemical modifications	Active packaging	[154]

CMC—carboxymethyl cellulose; CNF—cellulose nanofibers; PVA—polyvinyl alcohol; TEMPO—2,2,6,6-tetramethylpiperidine-1-oxyl.

The integration of aerogel-based bio-based hybrid packaging into the industry represents a breakthrough in addressing key challenges related to food preservation and sustainability.

The major applications and benefits of these materials for packaging are as follows (Table 3):

- Extended freshness and shelf life: the thermally insulating properties of aerogel-based bio-based hybrid packaging play a crucial role in regulating temperature fluctuations, a critical factor in the preservation of perishable goods. By creating a controlled and stable environment, these materials mitigate temperature fluctuations during storage and transport, extending the freshness and shelf life of food. This is particularly important for products that are sensitive to temperature fluctuations, such as fresh produce and dairy products.
- Reducing waste through improved packaging: A major reason for global waste is the inadequate preservation of perishable goods throughout the supply chain. Hybrid packaging based on bio-based aerogel acts as an effective barrier against temperature fluctuations, moisture, and external contaminants. By forming a protective cocoon for food, these materials significantly reduce the risk of premature spoilage. The result is a significant reduction in food waste as products have a longer shelf life. This is in line with sustainable practices and minimizes the environmental impact associated with discarded food.
- Improved product quality: The use of aerogel-based bio-based hybrid packaging goes beyond extending shelf life. These materials also help to preserve the quality and nutritional value of food and other products. The controlled microclimate created by the packaging ensures that products reach the consumer intact, maintaining strict quality standards and improving the overall consumer experience.
- Adaptability to different product types: The versatility of hybrid packaging based on bio-based aerogels allows them to be used in a variety of product categories. From fresh fruit and vegetables to temperature-sensitive dairy and meat products, these materials can be adapted to the specific requirements of different foods. This adaptability makes them a viable and sustainable choice for various segments of the food industry.

The use of bio-based hybrid packaging with aerogel technology in the packaging sector not only addresses the key challenge of extending freshness and minimizing food waste but also plays an important role in maintaining product quality and meeting the different requirements of different industry sectors. These materials represent a remarkable advance in sustainable packaging. They promote responsible practices and help build a robust and environmentally conscious product supply chain.

## 5. Scalability and Cost-Effectiveness

The application of bio-based aerogels in sustainable packaging requires a thorough assessment of scalability and cost-effectiveness, two critical factors for bringing breakthroughs from the lab to the industrial scale.

Scaling up the production of bio-based aerogels from the laboratory to industry poses a number of challenges. Maintaining the special properties of aerogels, such as their porosity and structural integrity, on a larger scale requires imaginative technical solutions. Addressing issues related to gelling, drying processes, and the use of environmentally friendly solvents is critical to the consistent and efficient production of bio-based aerogels in the quantities required for industrial packaging needs. On the other hand, cost efficiency is a key factor for the widespread adoption of bio-based aerogels for sustainable packaging. Optimizing every stage of production, from the extraction of raw materials to aerogel formation, is crucial. The introduction of novel processing techniques, such as continuous manufacturing processes, can minimize production costs [154,155,156,157,158]. Exploring the use of waste streams from other industries as feedstock for bio-based aerogels improves cost efficiency and is in line with the principles of the circular economy [157,158,159,160,161]. The choice of bio-based materials significantly influences overall production costs.

In addition to the inherent properties, availability, renewability, and regional availability also play a decisive role. Strategic choices, such as the use of agricultural residues or waste materials, not only reduce feedstock costs but also contribute to the valorization of by-products, thus promoting a sustainable and economically viable approach to bio-based aerogel production.

Energy-intensive processes in aerogel production can have a significant impact on costs. Evaluating and improving energy efficiency through innovative drying methods, such as microwave or supercritical drying, can reduce energy consumption. Process innovations, including the incorporation of green chemistry principles, can lead to more energy-efficient and cost-effective routes for the production of bio-based aerogels. The use of renewable energy sources is in line with sustainability goals while minimizing production costs. The quest for scalability and cost efficiency in bio-based aerogel production is an ongoing endeavor. Future research should focus on developing scalable and cost-efficient processing technologies, exploring alternative feedstocks, and optimizing overall supply chains. Innovations in automation and data-driven manufacturing can further increase efficiency. In addition, the continuous refinement of production processes for hybrid materials will bring bio-based aerogels to the forefront of sustainable packaging solutions.

Scalability and cost efficiency in the production of bio-based aerogels are crucial for their successful integration into the packaging industry. Overcoming scaling challenges, optimizing processes to increase efficiency, and fostering collaboration between academia and industry are essential steps to unlock the full potential of bio-based aerogels for sustainable packaging. Continued innovation and efficiency will undoubtedly help bio-based aerogels transform the environmental footprint of the packaging sector.

## 6. Conclusions and Future Prospects

In the search for sustainable packaging solutions, remarkable trends are emerging in the field of bio-based hybrid aerogels. Bio-based nanocomposite aerogels, which contain nanomaterials to enhance certain properties, represent a promising avenue. Ongoing research in this direction aims to improve the mechanical strength, thermal insulation, and barrier properties of hybrid aerogels. At the same time, there is a growing trend towards intelligent packaging systems that enable the real-time monitoring of product conditions. Innovations in sensor technologies, data analytics, and responsive materials can improve functionality and performance, expanding the scope of bio-based hybrid aerogels.

Research into hybrid materials composed of different bio-based aerogels is very promising for the development of sustainable packaging solutions. The development of hybrid bio-based aerogels for sustainable packaging is a dynamic field with promising future prospects. The objectives of this research included a thorough investigation of different production methods for hybrid materials from various bio-based aerogels for sustainable packaging. This investigation included a range of techniques combining bio-based aerogels with other materials to form hybrid composites that show the potential to optimize their properties for sustainable packaging applications. In addition, a key objective of this review was to evaluate the environmental friendliness and sustainability benefits of hybrid materials containing bio-based aerogels. The comprehensive assessment went beyond the synthesis process and looked at the environmental impact and long-term sustainability benefits associated with these innovative materials. As part of its forward-looking goals, the research went beyond the confines of the laboratory and aimed to discover practical applications of these hybrid materials for sustainable packaging. By identifying potential uses, such as improving insulation properties and barrier function, this study positions these hybrid materials as transformative agents in the ongoing pursuit of sustainable packaging practices. This multi-faceted approach emphasizes the importance of these goals and reinforces the role of hybrid materials as a viable solution to address the environmental challenges posed by traditional packaging methods.

The main challenge to the widespread adoption of bio-based hybrid aerogels for packaging is regulatory compliance. Although these materials represent sustainable and environmentally friendly alternatives, it is essential to navigate the complicated regulatory framework. Subsequent research efforts should focus on ensuring that bio-based hybrid aerogels not only meet but exceed existing standards for safety, labelling, and environmental compliance. Collaboration with regulators is essential to establish precise guidelines and certifications that instill confidence in manufacturers, consumers, and policy makers alike. The successful integration of aerogel-based bio-based packaging into the mainstream market depends on overcoming the barriers to market acceptance. Collaboration with industry players, including packaging manufacturers, retailers, and logistics providers, is critical. Building robust supply chains, optimizing production processes, and addressing cost factors are critical elements for market penetration. Strategic partnerships and knowledge sharing initiatives will accelerate the seamless integration of bio-based hybrid aerogels into the existing packaging ecosystem.

Further research is needed to explore novel crosslinking agents and reinforcement techniques that increase durability without compromising the environmentally friendly properties of these materials. In addition, investigating the scalability and cost-effectiveness of hybrid aerogel production is essential to eventually integrate them into mainstream sustainable packaging practices. The combination of different bio-based aerogels to produce hybrid materials, facilitated by crosslinking and reinforcement techniques, represents a transformative approach in the search for durable and sustainable packaging solutions. The careful selection of components, crosslinking agents, and reinforcing materials, as well as the consideration of environmental aspects, ensures that these hybrid materials make a positive contribution to the evolving landscape of environmentally friendly packaging technologies.

The outlined challenges and future directions for bio-based hybrid aerogels in packaging emphasize the multidimensional nature of their integration into the market. Regulatory compliance, market acceptance, and emerging trends are interrelated aspects that require a collaborative and interdisciplinary approach. By addressing these challenges and embracing new trends, the bio-based hybrid aerogel industry can not only fulfil the requirements for sustainable packaging but also pave the way for a greener and more resilient future in the packaging landscape.

## Figures and Tables

**Table 1:** Advantages and disadvantages of different raw materials for bio-based aerogel packaging materials [14,15,16,17,18,19,20,21,22,23,24,25,26,27,28,29,30,31,32,33,34,35,36,37,38,39,40,41,42,43,44,45,46,47,48,49,50,51,52,53,54,55,56,57,58,59,60,61,62,63,64,65,66,67,68,69,70,71,72,73,74,75,76,77,78,79,80,81,82,83,84,85,86,87,88,89,90,91,92,93,94,95,96,97,98,99,100,101,102,103,104,105,106,107,108,109,110,111,112].

Type of Raw Materials for Aerogel Production	Advantages	Disadvantages
Alginate-based aerogels	- biocompatible, suitable for different packaging applications; - mostly derived from brown algae, sustainable and renewable source for aerogel production; - are biodegradable, offering environmentally friendly disposal options at the end of their life cycle; - their production can be cost-effective due to the abundance of brown algae, potentially providing a more economical alternative to traditional aerogel materials.	- lower mechanical strength compared to synthetic counterparts, impacting their applicability in certain high-stress environments; - sensitive to moisture, affecting their stability and performance in humid conditions; - lower thermal stability compared to synthetic aerogels, restricting their use in applications requiring high-temperature resistance; - gelation and drying processes may require optimization for consistent and desirable properties and have limited resistance to certain chemicals.
Cellulose-based aerogels	- lightweight and high strength-to-weight ratio; - excellent thermal insulating properties; can be processed using eco-friendly methods; - biodegradable, renewable, and abundant resource, which reduces their carbon footprint; - cost-effective compared to some alternatives.	- limited mechanical strength compared to some alternatives; - need for additional treatments for optimal performance; - complex processing may pose challenges; - dependence on specific feedstock sources and energy-intensive processing methods.
Chitosan-based aerogels	- excellent adsorption capabilities, making them effective for removing pollutants, heavy metals, and other contaminants from liquids and gases; - biocompatible, sustainable, and renewable resource.	- lower mechanical strength compared to synthetic counterparts, affecting their structural integrity; - sensitive to high humidity, leading to potential degradation and reduced performance in humid conditions; - limited temperature resistance, and processing involves complex procedures; therefore, achieving uniform structures can be challenging, impacting scalability.
Lignin-based aerogels	- good thermal insulation properties; - sustainable and readily available raw material; - often a by-product of the paper and biofuel industries, reducing production costs for aerogels; - can be disposed of without harm to ecosystems.	- lignin sources can vary widely, leading to challenges in achieving consistent aerogel properties; - complex molecular structures, necessitating sophisticated processing methods; - may absorb moisture, impacting their long-term stability and performance; - may also exhibit brittleness, limiting their use in certain applications requiring flexibility.
Pectin-based aerogels	- easily modified to achieve a range of properties, enhancing their adaptability for various applications; - biocompatible, is a by-product of the fruit processing industry, making it cost-effective.	- varying properties depending on the source and extraction methods, leading to inconsistent performance; - sensitive to humidity and temperature, affecting their stability and performance in certain environments; - lower thermal and mechanical strength compared to synthetic aerogels; restricted use in high-temperature environments.
Protein-based aerogels	- excellent mechanical properties, lightweight, and with high porosity; - effective thermal insulation, suitable for diverse applications; - straightforward processing compared to some synthetic materials; - non-toxic and biocompatible, posing minimal health risks.	- varied performance depending on the protein source, requiring optimization; - limited availability of suitable protein sources for certain uses; - allergenic reactions possible depending on the protein source; - limited scalability and potential competition for food resources.
Starch-based aerogels	- non-toxic and pose fewer health risks during manufacturing and handling; - cost-effective, making them an economical choice for large-scale aerogel production; - biodegradable, contributing to environmental sustainability and reducing end-of-life concerns.	- lower mechanical strength; - sensitivity to moisture, potentially compromising the stability and performance of aerogels in humid conditions; - lower thermal stability, restricting their use in high-temperature applications; - the properties of starch-based aerogels can vary depending on the source and processing methods.

**Table 2:** Comparison of drying methods for preparation of hybrid bio-based aerogels [110,111,112,113,114,115,116,117,118,119,120,121,122,123,124,125,126,127,128,129,130,131,132,134,135].

Drying Method	Conditions	Preparations Prior to Drying Procedure	Advantages and Limitations
Freeze drying	Pressure under 100 mBar −80 °C < temperature < −40 °C	Use of additives or surfactants to modify the properties of the gel and prevent structural collapse during	Advantages: The removal of solvents from the gel via sublimation, preserving the porous structure; a controlled and uniform drying process; sublimation during freeze drying helps prevent shrinkage and cracking in the aerogel structure. Limitations: time-consuming process; increasing production costs; variations in ice crystal size during freezing can lead to structural irregularities within the aerogel that affect its mechanical and thermal properties.
Ambient drying	Room temperature Ambient pressure	Hydrophobization of the matrix; use of solvent that easily evaporates (water, alcohol, other organic solvents)	Advantages: Not-high costs; safe procedure. Limitations: not appropriate for fragile and hydrophilic matrices.
Supercritical drying	40 °C < temperature < 70 °C 70 Bar < Pressure < 200 Bar	Solvent should be compatible with CO_2_ (if used); no solvent conversion should occur during direct supercritical drying	Advantages: relatively fast, enabling efficient and time-saving production; removal of solvents without leaving residues, resulting in highly pure aerogel materials, occurs. Limitations: need for specialized high-pressure equipment and controlled environments presents limitation.
Microwave drying	40 °C < temperature < 80 °C Frequency: 2.45 GHz or 5.8 GHz	Composition of the gel precursor; ensuring a homogenous mixture of the gel precursor; proper stirring and mixing of the gel precursor solution to ensure homogeneity and uniform distribution of components.	Advantages: Acceleration of drying process; consistent drying and minimizing the risk of uneven structures or cracks in the aerogel; precise control of temperature and power. Limitations: occurs in microwaves, which can lead to rapid evaporation of volatile components, potentially affecting the composition and properties of the aerogel.

## Data Availability

Not applicable.
